# Causes of culling in dairy cows and its relation to age at culling and interval from calving in Shiraz, Southern Iran

**Published:** 2012

**Authors:** Maryam Ansari-Lari, Mehdi Mohebbi-Fani, Abbas Rowshan-Ghasrodashti

**Affiliations:** 1 *Department of Food Hygiene and Public Health, School of Veterinary Medicine, Shiraz University, Shiraz, Iran; *; 2 *Department of Animal Health Management, School of Veterinary Medicine, Shiraz University, Shiraz, Iran; *; 3 *Department of Internal Medicine, School of Veterinary Medicine, Islamic Azad University, Kazeroon Branch, Kazeroon, Iran.*

**Keywords:** Dairy herds, Cox model, Culling, Infertility, Iran

## Abstract

This study was designed to investigate causes of culling in industrial dairy herds in Fars province and to describe the pattern of reason-specific culling with respect to age of animal and interval from calving to culling. A total number of 9 dairy herds were selected for the study and information about culling reasons, birth date, last calving date and culling date was collected for culled cows during 2005-2006. Infertility (32.6% of all culls) was the most prevalent reason of culling followed by mastitis (6.5%). The time interval from last calving to culling averaged 240 days (SD = 176) and nearly 28% of cows were culled in the first 100 days after calving. Mean age of animals at culling was 6 years (SD = 2.7) and median was 5.7 years. In Cox proportional hazard model for calving to culling interval, infertility (hazard ratio [HR] = 0.26) showed lower risk whereas mastitis (HR = 2.40), left displaced abomasum (HR = 2.60) and peripartum problems (HR = 2.60) had higher risk of culling compared with voluntary cull. In the Cox model for age at culling, risk of culling was significantly higher for infertility (HR = 1.70), left displaced abomasum (HR = 3.15), and peripartum problems (HR = 2.10) compared with voluntary culling. In conclusion, farmers tend to keep infertile cows for longer period from calving to culling while infertile cows are generally culled at younger age. Also, early culling appeared to have a high proportion of culls in the studied herds.

## Introduction

Culling is the departure of cows from the herd due to sale, slaughter, or death. In general, culling has been classified as voluntary or involuntary.^1^ Involuntary culling implies that cows were culled due to disease, injury, infertility or death. Low yield or cows surplus to herd requirement are examples of voluntary culling when animals are healthy and farmer has complete freedom of choice over which cows are removed from the herd. An alternative conceptual distinction for culling has been to distinguish culling reasons as either biological or economic. Biological culls are those cows for which no possible productive future exists. Economic culls mean that cow is removed because a replacement is expected to produce greater profit.^2^ Optimum herd profitability is achieved by minimizing the proportion of the herd culled for involuntary or biological reasons and maximizing the proportion culled for voluntary or economic reasons.^3^

Identifying reasons for culling can be helpful in determining management problems in herds. From the epidemiologic point of view, description of the situation in each region is a prerequisite for every effort to be made to improve the understanding of importance and scope for sound production and reproduction management. Furthermore, there are important aspects of culling which have to be considered such as age at culling and interval from calving to culling. With identifying how these aspects may place an animal at increased risk of being prematurely removed from the herd, management practices can be better directed to minimize involuntary culling and increase herd profit.^3^ Limited studies have been conducted examining the culling in Iranian dairy herds.^4^^-^^6^ Therefore, the study presented here was designed to investigate causes of culling as stated by farmers or diagnosed by veterinarians in industrial dairy herds in Shiraz, the capital of Fars province, southern Iran and to describe the pattern of reason-specific culling with respect to age of animal and interval from calving to culling.

## Materials and Methods

This study was conducted in Fars province, southern Iran during March 2005 to September 2006. Target population was consisted of all Holstein dairy cows which were under registration of the dairy herd improvement program, by Agricultural Jihad Organization (AJO) of the province. A sample of 9 registered dairy farms was selected for the study based on the consent of the owners to participate. In the studied herds, the cows had non-seasonal reproductive programs and were bred routinely by artificial insemination (AI) done by a trained herd owner or an AI technician. Cows were milked three times per day and their rations were based primarily on corn silage, alfalfa hay and some grain mix. The farms had veterinary and nutrition consultants. 

Data about culling reasons were obtained prospectively using a prepared data sheet including the herd and cow identification, birth date, last calving date, culling date and 10 categories for culling reason (Table 1). Farmers were requested to fill out the sheets for the culled cows within the succeeding months. Completed forms were collected by each of the authors during the study period. The following data were calculated upon collection of the forms: age of animal at removal, age at last calving and time interval from last calving to culling. 

For statistical analysis, culled cows were grouped according to the reason of removal. Cox proportional hazard model was used and two separate Cox models were fitted using SPSS for Windows (Version 16.0, SPSS Inc., Chicago, IL, USA). In the first model, calving to culling interval was considered as dependent variable and removal categories, age at culling, season of birth and season of last calving were introduced into model as covariates. The hazard functions for culling with regard to interval from last calving to culling were compared for covariates in the model. In the second model, age at culling was considered as dependent variable and removal categories, season of birth and season of last calving were introduced into model as covariates. 

The outcome of interest was culling in both models and there was no censoring because only culled cows were included. Doing backward likelihood ratio elimination procedure and based on Wald statistics, final Cox models were fitted. In all analyses, a *p*-value less than 0.05 was considered as statistically significant difference.

## Results

The herd size in the studied farms varied from 30 to 750 milking cows. Out of 1235 animals, 269 cows (21.8%) were removed and 41 cows (3.3%) died during the study period. Overall culling rate was 25.1% in all herds for the study period. In Table 1, culled cows are shown according to the recorded reason. Among the involuntary causes of culling which comprised 74% of all culling, infertility (32.6% of all culls) was the most prevalent reason followed by mastitis (6.5%), peripartum problems (5.2%), left displacement of abomasum (LDA) (4.8%), physical injuries (4.2%) and lameness (3.5%). Miscellaneous causes (8.7%) were endocarditis, chronic diarrhea and/or emaciation, arthritis, traumatic reticuloperitonitis, respiratory problems and septicemia. Aging, low production, sale of animals due to financial needs and bad type (body conformation) as voluntary causes of culling was responsible for 26.0% of all culled cows. Among causes of death, most cases died of unknown causes followed by respiratory infections, peripartum health problems and mastitis.

**Table 1:** Frequency of various causes of culling (removed or dead) in 9 dairy herds in Fars province, Southern Iran (2005-2006).

**Reason of culling**	**No.**	**Percentage** **of all culls**	**Mean age** **(SD)**	**Median** **age**
***Voluntary causes***				
**Ageing**	25	8.1	10.1 (2.4)	10.9
**Body conformation**	13	4.2	5.5 (2.3)	5.4
**Financial needs**	16	5.2	5.2 (2.3)	4.2
**Low production**	27	8.7	5.2 (2.8)	4.7
				
***Involuntary causes***				
**Infertility**	101	32.6	6.1 (2.3)	5.7
**Lameness**	11	3.5	7.2 (1.9)	7.4
**LDA**	15	4.8	4.8 (2.5)	4.0
**Mastitis**	20	6.5	6.0 (2.8)	7.3
**Peripartum health problems**	16	5.2	6.0 (2.3)	6.0
**Physical injury**	13	4.2	5.3 (2.0)	4.7
**Miscellaneous causes** a	27	8.7	5.1 (2.4)	4.1
**Unknown reasons**	26	8.4	5.7 (2.1)	5.3
***Total***	310	100	6.1 (2.7)	5.7

a Including endocarditis, chronic diarrhea and/or emaciation, arthritis, traumatic reticuloperitonitis, respiratory problems and septicemia.

**Fig. 1:**
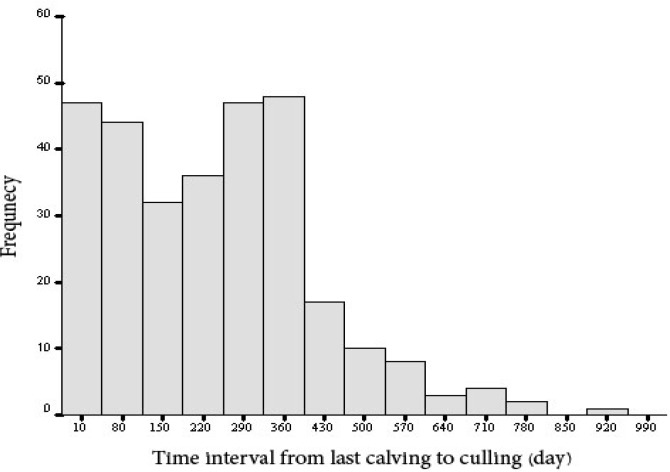
Frequency distribution of time interval from last calving to culling in 9 dairy herds in Fars province, Southern Iran (2005-2006).

Descriptive statistics for time interval from calving to culling according to causes of culling are presented in Table 2. The time interval from last calving to culling averaged 240 days with relatively large variation (SD = 176); the minimum time was 0 days due to peripartum problems and the maximum time was 948 days due to infertility. The distribution of calving to culling interval was bimodal with two peaks, up to 100 days after calving and then 290 to 360 days after calving (Fig. 1). Mean and median age of animals at culling was 6 (SD = 2.7) and 5.7 years, respectively. The first and third quartile of age at culling was 4 and 8 years, respectively. The oldest culled animal was 15 years old. Based on the cumulative frequency, 52.6% of the cows were removed by the end of fifth years of age. 

**Table 2:** Time interval (days) from last calving to culling (removed or dead) according to reasons in 9 dairy herds in Fars province, Southern Iran (2005-2006).

**Reason of culling **	**Mean**	**SD**	**Median**
**Infertility**	396	151	368
**Lameness**	145	133	95
**LDA**	85	96	32
**Mastitis**	100	103	73
**Miscellaneous causes** a	126	113	95
**Peripartum problems**	64	128	7
**Physical injury**	264	148	279
**Unknown causes**	257	133	236
**Voluntary reasons** b	187	114	192

a Including endocarditis, chronic diarrhea and/or emaciation, arthritis, traumatic reticuloperitonitis, respiratory problems and septicemia.

b Including ageing, low production, sale due to financial needs and bad type (body conformation).

Considering the time interval after last calving to culling, 40 animals (13.4%) were culled in the first 30 days after calving compared with 86.6% after 30 days. Similarly 28.4% were culled in the first 100 days after calving compared with 71.6% after 100 days. The proportions of voluntary causes of culling were 15.0% in the first 30 days and 22.0% in the first 100 days. Early culling reasons were mastitis, peripartum health problems and LDA. There was lower proportion of culling in autumn (15.0%) compared with spring, summer and winter that were 31.0, 31.0 and 21.0%, respectively (*p* < 0.001). 

When hazard of culling for calving to culling interval was compared according to various reasons of culling by Cox regression, infertility showed lower hazard. However, mastitis, LDA and peripartum health problems had higher hazard compared with voluntary culling. This indicated the longer time period from calving to culling for infertility compared with other reasons (Table 3). Hazard of culling was significantly lower for animals which calved in winter and summer compared with spring (Table 3). This implied that for each cow, the risk of culling in shorter interval from calving was greater if she calved in spring than in winter or summer. Season of birth and age at culling were dropped in the backward procedure.

**Table 3:** Estimates of hazard ratio for time from last calving to culling in 9 dairy herds in Fars province, Southern Iran (2005-2006).

**Factor**	**b**	**SE**	**HR**	**95% CI**	***p*** **-value**
***Season of calving ***					
**Spring**	-	-	-	-	-
**Summer**	-0.43	0.17	0.65	0.47-0.91	0.012
**Autumn**	-0.11	0.18	0.89	0.63-1.85	0.559
**Winter**	-0.33	0.16	0.72	0.52-0.99	0.040
***Reasons of culling***					
**Voluntary** a	-	-	-	-	-
**Infertility **	-1.35	0.17	0.26	0.18-0.36	< 0.001
**Lameness**	0.17	0.33	1.19	0.62-2.26	0.607
**Physical injury**	-0.72	0.31	0.49	0.27-0.89	0.021
**Mastitis **	0.87	0.26	2.40	1.43-4.04	0.001
**LDA**	0.95	0.29	2.59	1.46-4.61	0.001
**Peripartum problems**	0.97	0.29	2.64	1.49-4.70	0.001
**Miscellaneous ** b	-0.06	0.19	0.94	0.65-1.37	0.754

CI: Confidence interval.

a : Including ageing, low production, sale due to financial needs and bad type (body conformation).

b: Including diarrhea and/or emaciation, arthritis, traumatic reticuloperitonitis, respiratory problems, septicemia, metabolic diseases, and unknown reasons.

b : Regression coefficients; SE: Standard error; HR : Hazard Ratio;

When age at culling was dependent variable, hazard of culling was higher for infertility compared with voluntary culling. This indicated that infertile cows were generally culled in younger age than cows in voluntary group. Calving in summer and autumn was associated with decreased hazard of culling compared with calving in spring. Season of birth was excluded from the final model through backward procedure (Table 4). 

**Table 4:** Estimates of hazard ratio for age at culling in 9 dairy herds in Fars province, Southern Iran (2005-2006).

**Factor**	**b**	**SE**	**HR**	**95% CI**	***p*** **-value**
***Season of calving ***					
**Spring**	-	-	-	-	-
**Summer**	-0.51	0.16	0.60	0.44-0.83	0.002
**Autumn**	-0.58	0.19	0.56	0.38-0.82	0.003
**Winter**	-0.10	0.16	0.91	0.67-1.24	0.544
***Reasons of culling***					
**Voluntary** a	-	-	-	-	-
**Infertility **	0.51	0.16	1.66	1.21-2.27	0.002
**Lameness**	0.39	0.33	1.47	0.77-2.82	0.243
**Physical injury**	0.98	0.31	2.67	1.45-4.92	0.002
**Mastitis **	0.49	0.27	1.64	0.97-2.77	0.064
**LDA**	1.15	0.31	3.15	1.72-5.77	<0.001
**Peripartum problems**	0.74	0.30	2.10	1.18-3.74	0.012
**Miscellaneous** b	0.88	0.20	2.41	1.62-3.57	<0.001

CI: Confidence interval.

a : Including ageing, low production, sale due to financial needs and bad type (body conformation).

b : Including diarrhea and/or emaciation, arthritis, traumatic reticuloperitonitis, respiratory problems, septicemia, metabolic diseases, and unknown reasons.

b : Regression coefficients; SE: Standard error; HR : Hazard Ratio

## Discussion

In the present study like most other works around the world, the most prevalent cause of culling dairy cows was infertility or failure to conceive.^3^^,^^4^^,^^7^^-^^10^ Causes for infertility in dairy cattle are of multifactorial origin and many factors such as management, nutrition and genetics are among the contributing factors.^11^ Low heritability estimates for reproductive factors suggest that genetic factors may explain only a small proportion of variation in fertility.^12^^,^^13^ It means that it is unlikely that all of the cows culled for infertility were really infertile;^8^ and improved nutrition, estrus detection and overall reproduction management may reduce the incidence of culling and associated costs due to infertility.

Based on statistical results, HR was significantly lower for infertility compared with voluntary culling with respect to calving to culling interval (Table 3). However, when age of animal was included as the dependent variable in the Cox regression model, infertility showed higher risk of culling than voluntary group. This implies that the farmers tend to keep infertile but otherwise healthy cows for a long time after calving before they decide to cull them while, in the same time, infertile culled cows are generally younger than animals culled voluntarily. This finding is consistent with a previous study which showed that culled cows for infertility were removed from the herd in younger age but later within lactation compared with udder disorders or voluntary reasons.^14^ Because farmers generally cull low producing cows, it is suggested that only high producing infertile cows skip from culling for longer periods of time. Nevertheless, profitability decreases with increasing days in milk for open cows.^14^ Therefore, education of farmers for implementation of better management policies as well as for correction of their attitudes toward culling could be beneficial to reduce cost of culling.

Mastitis was the second most prevalent reason of culling as previously reported by others.^8^^,^^9^^,^^14^^,^^15^ Most cases of mastitis were associated with early culling. This is in agreement with Seegers *et al.* while is not consistent with finding of Dohoo and Martin which reported a significant association of mastitis with late culling.^14^^,^^16^ Also, Rajala-Schultz and Gröhn showed that mastitis had a significant effect on culling throughout the whole lactation, however, they indicated that at the end of lactation (>240 days after calving), mastitis seemed to have a protective effect with the risk of the cow being culled.^15^

Regarding age at culling, no significant difference was observed between voluntary culled cows and cows which were culled due to mastitis. This could be explained by the fact that incidence of mastitis is increased with increasing age and parity of animal.

Peripartum health problems and LDA were reasons which resulted in early culling (within the first 30 or 100 days in milk) of animals like mastitis in the present study. This is similar to previously reported results in which days in milk were 1 to 30 and 100 days, for LDA and peripartum health events, respectively.^14^^,^^17^ While the goal is to keep the number of early culling as low as possible, early culling was responsible for more than one fourth of culls (28.0%) in the present study. In addition, it is suggested by Salfer that almost all cows that leave the herd during the first 100 days in milk should be involuntary culls. In contrast to this suggestion, 22.0% of all culled cows in this period were voluntary culls in the present study. Most of the early lactation culled cows can be traced to improper management during the transition period^18^ and improved management particularly nutrition as well as better udder health decreases this category of culling and its subsequent economic losses. With respect to age at culling, LDA and peripartum health problems showed significantly higher risk of culling compared with voluntary culls. 

Bimodal distribution of calving to culling interval was observed in our study reported previously by Stevenson and Lean and Seegers *et al*.^3^^,^^14^ This finding indicates that if animal had the chance to remain in the herd within 100 days after calving, the second most hazardous period for her culling would be around the 10-12 months later. Therefore, the end of lactation appeared to be an important period for farmers to decide if a cow was to remain in the herd.^3^ The main reasons for culling cows within this period were infertility, lameness, voluntary causes and physical injuries in the present study. 

For the association between season of calving and risk of culling, we found that hazard of culling was significantly lower for animals which calved in winter and summer compared with spring months. This may be attributed to the attitudes of farmers toward culling in different seasons which is based on predictable climatic changes. Spring is followed by summer with increasing heat stress whereas winter and summer are followed by spring and autumn, respectively, with improving climatic conditions. Therefore, it is concluded that farmers tend to cull diseased cow in spring to avoid a worsening condition in summer. Instead they could keep her in summer and winter to see if she is recovered from the disease with improving environmental conditions. 

In conclusion, the main reason for culling in our study herds were infertility followed by diseases particularly mastitis. While farmers tend to keep infertile cows for longer period from calving to culling, infertile cows are generally younger than voluntarily culled animals. Also, while the goal is to keep the number of early culling as low as possible, early culling was responsible for more than one fourth of culls (28.0%) in the present study. Therefore, adoption of higher standards of management and husbandry with respect to fertility and other health disorders could prevent involuntary culling and reduce the cost of premature losses of animals. Exploring the attitudes of farmers toward culling and improvement of incorrect perceptions through planning appropriate educational programs is also warranted.
